# Outcomes of high BMI colorectal cancer patients with natural orifice specimen extraction surgery: a propensity-score matching study

**DOI:** 10.3389/fsurg.2025.1624266

**Published:** 2025-08-21

**Authors:** Chenkai Zhang, Hao Zhang, Wenyang Li, Songtao Yu, Haonan Qi, Chunlin Wang, Ibatullin Artur, Guiyu Wang

**Affiliations:** ^1^ Department of Colorectal Surgery, The Second Affiliated Hospital of Harbin Medical University, Harbin, China; ^2^ Department of Ophthalmology, the Future Medicine Laboratory, The Second Affiliated Hospital of Harbin Medical University, Harbin, China; ^3^ Bashkir State Medical University, Ufa, Russia; ^4^ Department of Colorectal Surgery, Zhejiang Cancer Hospital, Hangzhou Institute of Medicine (HIM), Chinese Academy of Sciences, Hangzhou, Zhejiang, China

**Keywords:** colorectal cancer, noses, BMI, outcomes, surgery

## Abstract

**Background:**

Natural orifice specimen extraction surgery (NOSES) is widely used for colorectal cancer. However, there is limited study regarding the outcomes of patients with high BMI who undergo NOSES surgery for colorectal cancer.

**Methods:**

This retrospective study included 251 patients (including 205 Non-High BMI and 46 High BMI patients) who underwent NOSES for colorectal cancer between January 2013 and December 2018. Outcomes related to surgery, anal function and long-term survisval were compared between the Non-High BMI and High BMI patients with the propensity-score matching (PSM) method. Age, gender, tumor location (sigmoid/rectum), preoperative CEA, CA199, T stage and N stage were considered as covariates for PSM.

**Results:**

After matching, 44 patients in the Non-High BMI group and 44 patients in the High BMI group were eligible for analysis. No significant differences were observed between the groups in terms of operative time, blood loss, time to first flatus, time to first diet, postoperative hospital stays, positive margin, postoperative complication, conversion to open surgery and pathological outcomes (all *P*-value > 0.05). Besides, there was no significant difference for anal function 6 months after surgery between the two groups (*P* = 0.723). The overall survival (OS) and disease-free survival (DFS) for the Non-high BMI group were comparable to those for the High BMI group (*P* = 0.156 for OS, *P* = 0.266 for DFS).

**Conclusion:**

With careful preoperative evaluation, High-BMI patients can successfully undergo NOSES surgery and achieve favorable outcomes.

## Introduction

Colorectal cancer is one of the most common malignant tumors worldwide. It is the third most common cancer in terms of incidence and the second most common in terms of mortality, posing a significant threat to human health (1). Laparoscopic-assisted natural orifice specimen extraction surgery (NOSES) for colorectal cancer was first reported by Franklin in 1993 (2). NOSES has been shown to outperform conventional laparoscopic colectomy in terms of cosmetic outcomes, shorter hospital stays, and reduced postoperative pain, making it an attractive minimally invasive procedure for colorectal cancer (3–5). In NOSES, the specimen is extracted through a natural orifice (anus or vagina), and the digestive tract is reconstructed within the abdominal cavity. The procedure leaves only minimal trocar scarring, embodying the concept of minimally invasive surgery with a true unassisted abdominal incision.

Higher body mass index (BMI) can impact the surgical procedure and prognosis. Some studies have shown that a high BMI adversely affects laparoscopic colorectal surgery, leading to poorer prognostic outcomes for patients (6). However, other studies have found no significant association between high BMI and short-term prognosis after surgery (7). Most of these studies have focused on the effects of BMI on conventional laparoscopic surgery for colorectal cancer, with limited research on its impact in NOSES surgery. Furthermore, BMI is considered an important factor in screening patients for NOSES eligibility. However, based on our extensive experience, we have observed that BMI cannot fully represent visceral fat, and is not a definitive factor influencing the feasibility of NOSES surgery. Many patients with high BMI after careful intraoperative assessment of indications, have successfully undergone NOSES surgery. However, there remains a gap in evaluating the safety and efficacy of NOSES surgery in high-BMI patients.

Therefore, the aim of this study was to compare the short-term and long-term outcomes between Non-High BMI and High BMI patients undergoing NOSES surgery for colorectal cancer, using propensity score matching (PSM) analysis.

## Methods and materials

The reporting and interpretation of this comparative effectiveness study followed the Strengthening the Reporting of Observational Studies in Epidemiology (STROBE) (8).We retrospectively selected patients diagnosed with sigmoid colon or rectal cancer who underwent NOSES surgery at the Second Hospital of Harbin Medical University between January 2013 and December 2018.All operations were performed by the same surgical team.The inclusion criteria were as follows: (1) pathological diagnosis of stage I-III sigmoid colon or rectal malignant tumors; (2) patients aged ≥18 years; (3) tumors located ≤30 cm from the anal verge; and (4) tumor diameter <5 cm. Exclusion criteria included: (1) patients receiving neoadjuvant therapy; (2) patients undergoing emergency surgery due to hemorrhage, obstruction, or perforation; (3) patients with a protective ostomy; (4) patients with multiple primary colorectal cancers; (5) incomplete clinical or pathological data; and (6) patients with distant metastases.

Patients were categorized into a Non-high BMI group (BMI < 25 kg/m^2^) and a High BMI group (BMI ≥ 25 kg/m^2^), based on previous studies (9, 10). PSM was applied to compare the perioperative and long-term clinical outcomes between the two groups. The covariates matched were age, gender, tumor location (sigmoid colon vs. rectum), clinicopathological stage, and preoperative levels of CEA and CA199.

### Surgery

Intra-abdominal resection and specimen extraction(IREX): For method one in the NOSES surgery, the procedure was as follows. The distal rectum below the tumor was transected with an ultrasonic scalpel and the intestine ends were sterilized with povidone gauze. After that, a disposable sterile protective cover was inserted into the abdominal cavity via the 12 mm trocar, with one end positioned in the opened rectal stump, and the other end pulled out of the anus, and then the anvil was introduced into the abdominal cavity through the protective cover. An incision was made above the tumor in the proximal colon wall, and the anvil was introduced into the colon through the incision. The proximal colon was closed using linear cutter-straight, leaving the anvil head extracted from the bowel lumen. The isolated specimen was placed into the protective cover and extracted from the pelvic cavity through the rectum. A linear stapler was used to close the rectal stump and the additional rectal section was placed into a specimen pouch and extracted via the 12 mm trocar. Finally, an end-to-end colorectal anastomosis was completed.

Specimen extraction and extra-abdominal resection (EXER): For method two in the NOSES surgery, the procedure was as follows. The rectal wall below the tumor was transected with an ultrasonic scalpel and a protective cover was placed into the pelvic cavity via the anus. A long grasper was introduced into the lumen through the anus to pull the specimen outside of the body gently and the specimen was removed extraabdominally. Then, the anvil was placed in the sigmoid colon and closed with a purse string and the bowel lumen was reintroduced into the abdominal cavity. After releasing the pneumoperitoneum, a linear stapler was used to close the open rectal stump and an end-to-end colorectal anastomosis was performed.

Specimen eversion and extra-abdominal resection (EVER): For method three in the NOSES surgery, the procedure was as follows. After the anal canal was dilated gently, the protective cover was inserted into the rectum. Then, the anvil was placed into the bowel lumen, until to the proposed resection line of sigmoid colon. The proximal colon division was performed, leaving the anvil inside of proximal bowel. A clamp was used to grab the rectal stump and to drag it out extracorporeally. The reversed rectum was fully disinfected and resected extra abdominally. Finally, the rectum was delivered back to patient body and an end to-end anastomosis was performed.

### Data collection

We performed a retrospective review of operative time, blood loss, time to first flatus, time to first diet, postoperative hospital stays, histologic type, number of lymph nodes harvested, positive margins, and postoperative complications. The Wexner score was used to evaluate the severity of fecal incontinence (11–13). Using this easy-to-use scale, anal function was compared between patients in the Non-high BMI and High BMI groups at 6 months after surgery.The pathologic stage was categorized as per the American Joint Committee on Cancer (AJCC) 8th edition. Postoperative complications were graded according to the Clavien–Dindo classification (14).

In our department, patients were followed up every 3 months for the first 2 years, every 6 months for the subsequent 3 years, and annually thereafter. Blood tests (CEA and CA199), physical examinations, and CT scans (including chest, abdomen, and pelvis) were performed at each follow-up. Colonoscopy was performed annually. Long-term tumor prognosis was assessed in terms of overall survival (OS) and disease-free survival (DFS).

### Data analysis

All statistical analyses were conducted using SPSS for Windows version 27.0. To minimize selection bias, we used the covariates of gender, tumor location, age, T-stage, N-stage, and CEA/CA199 levels for 1:1 propensity-score matching (PSM) with a caliper value of 0.2. Data from patients after PSM matching were used for further analysis. Qualitative data were compared using the chi-square test or Fisher's exact test. For continuous data, the Mann–Whitney *U* test (for non-normally distributed values) or independent *t*-test (for normally distributed values) was used. Kaplan–Meier curves were plotted for OS and DFS and analyzed using the log-rank test. A *p*-value < 0.05 (two-sided) was considered statistically significant.

## Results

### Baseline data for the non-high BMI and high BMI groups

In our retrospective analysis, 205 patients in the Non-high BMI group and 46 patients in the High BMI group met the inclusion and exclusion criteria. Before PSM, significant differences were observed between the groups in terms of CA199 (*P* = 0.004) and N-stage (*P* = 0.001). After 1:1 PSM matching, the following variables were successfully balanced: age (*P* = 0.588), tumor location (*P* = 0.796), gender (*P* = 0.842), T-stage (*P* = 0.573), N-stage (*P* = 0.969), CEA levels (*P* = 0.437), and levels CA199 (*P* = 0.777) (Table 1).

**Table 1:** Baseline characteristics of patients.

Characteristic	After PSM			After PSM	After PSM	After PSM
	Non-high BMI(*n* = 205)	High BMI (*n* = 46)	*P*	Non-high BMI(*n* = 44)	High BMI (*n* = 44)	*P*
Age(years)^a^	59.72 ± 11.8	59.93 ± 10.7	0.911	58.66 ± 10.7	59.91 ± 11.0	0.588
Gender						
Male	95 (46.3)	16 (34.8)	0.154	19 (43.2)	16 (36.4)	0.842
Female	110 (53.7)	30 (65.2)		25 (56.8)	28 (63.6)	
Tumor location						
Sigmoid	43 (21)	10 (21.7)	0.909	10 (22.7)	9 (20.5)	0.796
Rectum	162 (79)	36 (78.3)		34 (77.3)	35 (79.6)	
Preoperative CEA						
Negative	168 (82)	33 (71.8)	0.117	36 (81.9)	33 (75)	0.437
Positive	37 (18)	13 (28.2)		8 (18.9)	11 (25)	
Preoperative CA199						
Negative	192 (93.7)	37 (85.5)	**0**.**004**	36 (81.9)	37 (84.1)	0.777
Positive	13 (6.3)	9 (14.5)		8 (18.9)	7 (15.9)	
T stage						
Tis	7 (3.4)	0 (0)	0.615	0 (0)	0 (0)	0.573
T1	34 (16.6)	7 (15.2)		4 (9.1)	6 (13.6)	
T2	51 (24.9)	11 (23.9)		6 (13.6)	10 (22.7)	
T3	75 (36.6)	21 (45.7)		26 (59.1)	21 (47.7)	
T4	38 (18.5)	7 (15.2)		8 (18.2)	7 (15.9)	
N stage						
N0	153 (74.6)	21 (45.7)	**0**.**001**	22 (63.5)	21 (47.7)	0.969
N1	41 (20)	20 (43.5)		17(28.8)	19(43.2)	
N2	11(5.4)	5(10.8)		5(7.7)	4(9.1)	

^a^ Mean ± SD. Bold marked figures are for variables with *P* < 0.05.

### Intraoperative and perioperative outcomes

There was no significant difference in operative time between the Non-high BMI and High BMI groups (186.18 ± 45.6 min vs. 192.98 ± 53.88 min, *P* = 0.726). Similarly, there was no significant difference in estimated blood loss between the two groups (51.93 ± 62.22 ml vs. 48.64 ± 40.56 ml, *P* = 0.643). No conversions to open surgery were required in either group. The time to first flatus did not differ significantly between the Non-high BMI and High BMI groups (49.77 ± 24.15 h vs. 51.14 ± 24.16 h, *P* = 0.766). Additionally, there was no significant difference in the time to first diet (66.25 ± 27.01 h vs. 70.32 ± 39.87 h, *P* = 0.748). Postoperative hospital stay was also similar between the two groups (12.45 ± 5.53 days vs. 14.75 ± 9.06 days, *P* = 0.217).

The High BMI group had a slightly higher rate of postoperative complications, but this difference was not statistically significant (4.7% vs. 9.1%, *P* = 0.676) and the morbidity grading was roughly similar in both groups(rated according to the Clavien–Dindo Classification of Surgical Complications). In the Non-high BMI group, one patient suffered from anastomotic leakage and one patient suffered from wound-related complication. In the High BMI group, three patients suffered from anastomotic leakage and one patient suffered from intra-abdominal abscess, as detailed in (Table 2).

**Table 2:** Intraoperative and perioperative outcomes in Non-high BMI group and high BMI group.

Characteristic	Non-high BMI (*n* = 44)	High BMI (*n* = 44)	*P*
Operative time (min)^a^	186.18 ± 45.6	192.98 ± 53.88	0.726
Blood loss (ml)^a^	51.93 ± 62.22	48.64 ± 40.56	0.643
Time to first flatus (h)^a^	49.77 ± 24.15	51.14 ± 24.16	0.766
Time to first diet (h)^a^	66.25 ± 27.01	70.32 ± 39.87	0.748
Postoperative hospital stays (days)^a^	12.45 ± 5.53	14.75 ± 9.06	0.217
Positive margin	0	0	–
Postoperative complication	2 (4.7)	4 (9.1)	0.676
Anastomotic leak	1 (2.3)	3 (6.8)	0.616
Anastomotic bleeding	0 (0.0)	0 (0.0)	–
Postoperative ileus	0 (0.0)	0 (0.0)	–
Intra-abdominal abscess	0 (0.0)	1 (2.3)	1.000
Pneumonia	0 (0.0)	0 (0.0)	–
Wound-related complication	1 (2.3)	0 (0.0)	1.000
Conversion to open surgery	0 (0.0)	0 (0.0)	–
Grade of morbidity (%)			
Dindo I–II	1 (2.3)	1 (2.3)	1.000
Dindo III–IV	1 (2.3)	3 (6.8)	0.616

^a^ Mean ± SD. Bold marked figures are for variables with *P* < 0.05.

**Figure 1:**
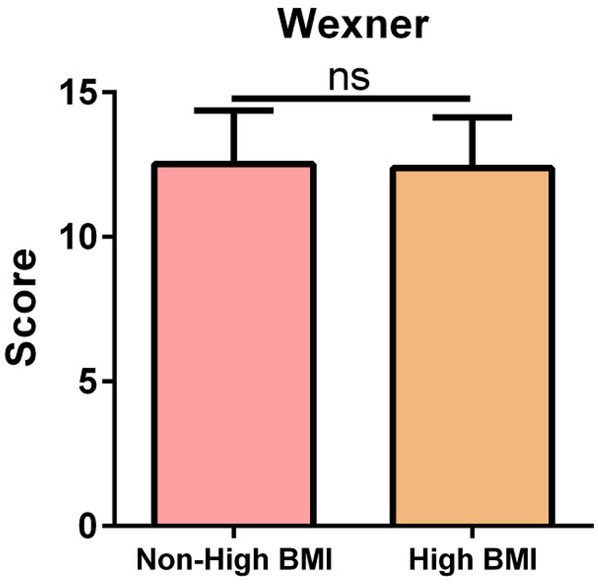
Comparison of anal function. ns, no significant difference.

Regarding anal function at 6 months postoperatively, there was no significant difference in the Wexner incontinence scale scores between the two groups (*P* = 0.723) (Figure 1).

Among the 88 patients included in the study, 42 (47.7%) were assigned to the EXER group, 26 (29.5%) to the EVER group, and 20 (22.7%) to the IREX group. Perioperative analysis demonstrated that the IREX group had a slightly shorter operative time compared to the other two groups; however, the difference was not statistically significant (*P* = 0.734). No significant differences were observed in estimated blood loss (*P* = 0.296) or the number of lymph nodes dissected (*P* = 0.208) among the three groups. Postoperative recovery parameters, including time to first flatus (*P* = 0.716), time to resumption of a regular diet (*P* = 0.984), and length of hospital stay (*P* = 0.979), also showed no significant intergroup differences.The overall postoperative complication rate was 8.0%, with 11.9% in the EXER group, 3.8% in the EVER group, and 5.0% in the IREX group; these differences were not statistically significant (*P* = 0.300) (Supplementary Table S1).

### Pathology and oncology results

**Figure 2:**
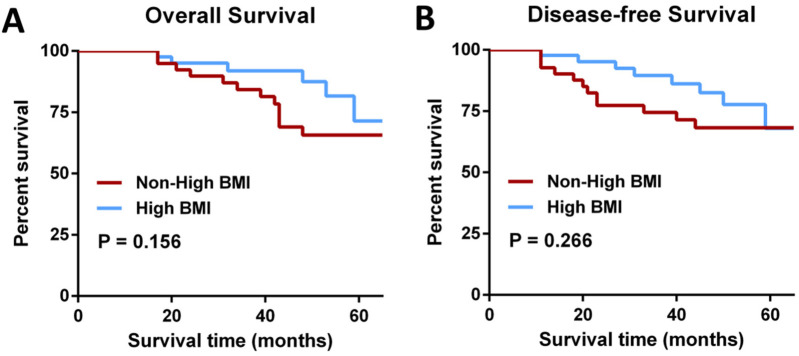
Survival comparisons. **(A) Overall survival** (log-rank *P* = 0.156; HR = 0.521, 95% CI 0.195–1.38, *P* = 0.192). **(B) Disease-free survival** (log-rank *P* = 0.266; HR = 0.457, 95% CI 0.175–1.218, *P* = 0.118).

The number of lymph nodes removed was similar in both groups (13.55 ± 4.48 vs. 13.07 ± 4.92, *P* = 0.691). Pathological data analysis showed that the tumor grading of patients in the Non-high BMI group and High BMI group was similar (*P* = 0.426), and most of the postoperative histological differentiation of patients in the two groups was moderate, and the differentiation was also balanced between the groups (*P* = 0.484) (Table 3). The overall survial in the Non-high BMI group were comparable to those in the High BMI group (log-rank *P* = 0.156; HR = 0.521, 95% CI:0.195–1.38, *P* = 0.192), and there was no significant difference in disease-free survival curves between the two groups (log-rank *P* = 0.266; HR = 0.457, 95% CI:0.175–1.218, *P* = 0.118) (Figure 2). Pathological assessment within the surgical subgroups revealed a comparable distribution of tumor grades among the groups (*P* = 0.273). Furthermore, the majority of cases exhibited moderate histological differentiation across all groups (*P* = 0.214) (Supplementary Table S1). Long-term prognostic analysis among the surgical subgroups revealed no statistically significant differences in Overall Survival (OS) between the EXER group and the EVER group (*P* = 0.281), or between the EXER group and the IREX group (*P* = 0.200). Similarly, no significant difference in OS was observed between the EVER and IREX groups (*P* = 0.495). Regarding Disease-Free Survival (DFS), the EXER group did not show a statistically significant difference compared to either the EVER group (*P* = 0.091) or the IREX group (*P* = 0.571). Likewise, the DFS difference between the EVER and IREX groups was not statistically significant (*P* = 0.356) (Supplementary Figure S2).

**Table 3:** Pathologica and oncologic outcomes in Non-high BMI group and high BMI group.

Characteristic	Non-high BMI (*n* = 44)	High BMI (*n* = 44)	*P*
Harvested lymph nodes^a^	13.55 ± 4.48	13.07 ± 4.92	0.691
Tumor grade			
Well-differentiated	6 (13.6)	3 (6.8)	0.426
Moderately differentiated	31 (70.5)	36 (81.8)	
Poorly differentiated	7 (15.9)	5 (11.4)	
Histological type			
Adenocarcinoma	36 (81.8)	39 (88.6)	0.484
Mucinous/Signet-ring cell	1 (2.3)	0 (0.0)	
Others	7 (15.9)	5 (11.4)	

^a^ Mean ± SD. Bold marked figures are for variables with *P* < 0.05.

## Discussion

After more than a decade of research and development, NOSES surgery has become widely used for colorectal tumors (15). Several studies have investigated the impact of different BMI grouping values on adverse events during lumpectomy (16, 17). With extensive experience in performing NOSES colon resections, our center analyzed clinical data from colorectal cancer patients who underwent NOSES surgery between January 2013 and December 2018. Short-term outcomes and long-term oncologic results in Non-high BMI and High BMI groups were assessed using propensity score matching (PSM) to minimize baseline data bias in retrospective controlled studies.

BMI is a commonly used, objective measure of body fat. According to the World Health Organization (WHO), a normal BMI falls between 18.5 kg/m^2^ and 25 kg/m^2^, so we categorized patients into a Non-high BMI group and a High BMI group. Tsujinaka et al. found that the visceral adiposity index (VAI) was more effective than BMI in predicting surgical outcomes after laparoscopic colorectal surgery (18, 19). However, BMI remains a more accessible and practical tool for estimating body fat, particularly in developing countries like China.

In the existing literature, high BMI has been shown to impact laparoscopic surgery and patient prognosis, with reports indicating longer operative times in high BMI patients (20). In contrast, our study found no significant difference in operative times between the Non-high BMI and High BMI groups in NOSES surgery (*P* = 0.726). Interestingly, one study reported a shorter mean operative time in obese patients, although the difference was not statistically significant (21). Numerous studies conducted in Western countries have examined the impact of high BMI on postoperative complications in laparoscopic colorectal cancer surgery, with mixed and controversial results (22). Similarly, in our NOSES surgery cohort, we observed a slightly higher incidence of overall postoperative complications in the High BMI group (4.7% vs. 9.1%, *P* = 0.676), but this difference was not statistically significant. Our findings align with several high-quality clinical studies, which showed broadly similar perioperative outcomes, including complications such as anastomotic fistula, bleeding, and intestinal obstruction, in both BMI groups (9, 23–25).The lack of significant differences among the EVER, EXER, and IREX surgical subgroups in surgical procedure, short-term outcomes, or long-term prognosis indicates that surgical technique did not confound the analyses comparing the non-high BMI and high BMI groups.

Moreover, even when specimens were extracted via the anus, the recovery of postoperative anal function was satisfactory in both groups. We used the Wexner incontinence scale scores to assess preoperative and postoperative anal sphincter function. There was no significant difference in the Wexner incontinence scale scores between the groups.

Our long-term follow-up demonstrated that survival outcomes and tumor safety were comparable between the Non-high BMI and High BMI groups. Pathological outcomes in both groups were also similar, with no statistically significant differences, which supports the findings of previous studies (19). Despite the increasing adoption of NOSES surgery for colorectal cancer, many guidelines recommend caution about its use in obese patients (26). Patients with a high BMI may have a large amount of visceral fat, which can lead to difficulties in removing specimens through natural orifices. Besides, increased visceral and abdominal wall fat in some high BMI patients can limit surgical space and then increase technical challenges, often leading to longer operative times in laparoscopic procedures (27). In our study, higher BMI did not significantly extend operative time in NOSES surgery nor did it increase the incidence of postoperative complications. These favorable outcomes are likely attributable to the extensive experience and stability of our surgical team. What is more, based on our team's extensive experience in NOSES (28–30), it has been found that, through further intraoperative assessment, a considerable number of patients with a high BMI are still suitable for undergoing NOSES surgery. If these patients are unable to receive NOSES surgery simply due to the preoperative BMI assessment, they will be deprived of the benefits that NOSES surgery can bring. The results of our study have provided objective evidence for the feasibility of applying NOSES in patients with a high BMI.

Still, our study has some limitations. First, as a retrospective study, selection bias is inevitable, which may reduce the generalizability of the findings. To minimize selection bias, we applied PSM to balance baseline characteristics and ensure a more objective and comprehensive analysis. Second, NOSES is an emerging technique with stringent indications, and the sample size in this study was relatively small. We grouped patients into Non-high and High BMI categories, rather than using multiple BMI subgroups as in previous studies (7, 9, 31). Third, although the current study suggested that patients with high BMI might also benefit from NOSES, surgeons should still strictly adhere to the indications strictly adhere to the indications to ensure the success rate of NOSES for patients with a high BMI. Moreover, we have not developed an objective and quantitative assessment method to determine which high BMI patients are suitable for NOSES. Fourth, the application of standardized exclusion criteria (e.g., no distant metastases, tumor diameter ≤5 cm, no severe adhesions) optimized feasibility and safety within this cohort but inherently limits the applicability of our findings to patients with advanced disease, larger tumors, or complex anatomy. Fifth, the technical complexity of NOSES demands advanced surgical skills; thus, the favorable outcomes observed likely reflect our team's specialized expertise and may not be generalizable to centers without comparable experience. Finally, as a single-institution study, our conclusions are inevitably influenced by institution-specific protocols, demographics, and practices. To address these limitations—particularly concerns regarding generalizability, the need for objective selection criteria, and center-specific factors—and to enhance the external validity of the results, future large-scale, multicenter prospective studies are needed.

## Conclusions

In conclusion, while BMI is an important factor during patient screening for NOSES surgery, it is not a determining factor for the success of the procedure. With careful preoperative evaluation, NOSES surgery for colorectal cancer might be both feasible and safe for CRC patients with high BMI.

## Data Availability

The raw data supporting the conclusions of this article will be made available by the authors, without undue reservation.

## References

[1] Siegel RL Miller KD Goding Sauer A Fedewa SA Butterly LF Anderson JC Colorectal cancer statistics, 2020. CA Cancer J Clin. (2020) 70(3):145–64. 10.3322/caac.21601 32133645

[2] Franklin ME Ramos R Rosenthal D Schuessler W. Laparoscopic colonic procedures. World J Surg. (1993) 17(1):51–6. 10.1007/BF01655705 8447141

[3] Wolthuis AM Fieuws S Van Den Bosch A van Overstraeten A dB D’Hoore A. Randomized clinical trial of laparoscopic colectomy with or without natural-orifice specimen extraction. Br J Surg. (2015) 102(6):630–7. 10.1002/bjs.9757 25764376

[4] Li XW Wang CY Zhang JJ Ge Z Lin XH Hu JH. Short-term efficacy of transvaginal specimen extraction for right colon cancer based on propensity score matching: a retrospective cohort study. Int J Surg Lond Engl. (2019) 72:102–8. 10.1016/j.ijsu.2019.07.025 31362128

[5] Zhang M Hu X Guan X Zheng W Liu Z Jiang Z Surgical outcomes and sexual function after laparoscopic colon cancer surgery with transvaginal versus conventional specimen extraction: a retrospective propensity score matched cohort study. Int J Surg Lond Engl. (2022) 104:106787. 10.1016/j.ijsu.2022.106787 35922001

[6] Hede P Sörensson MÅ Polleryd P Persson K Hallgren T. Influence of BMI on short-term surgical outcome after colorectal cancer surgery: a study based on the Swedish national quality registry. Int J Colorectal Dis. (2015) 30:1201–7. 10.1007/s00384-015-2280-0 26077669

[7] Hirpara DH O’Rourke C Azin A Quereshy FA Wexner SD Chadi SA. Impact of BMI on adverse events after laparoscopic and open surgery for rectal cancer. J Gastrointest Cancer. (2022) 53(2):370–9. 10.1007/s12029-021-00612-2 33660225

[8] von Elm E Altman DG Egger M Pocock SJ Gøtzsche PC Vandenbroucke JP The strengthening the reporting of observational studies in epidemiology (STROBE) statement: guidelines for reporting observational studies. Lancet Lond Engl. (2007) 370(9596):1453–7. 10.1016/S0140-6736(07)61602-X 18064739

[9] Poulsen M Ovesen H. Is laparoscopic colorectal cancer surgery in obese patients associated with an increased risk? Short-term results from a single center study of 425 patients. J Gastrointest Surg. (2012) 16(8):1554–8. 10.1007/s11605-012-1928-0 22688417

[10] Park JW Lim SW Choi HS Jeong SY Oh JH Lim SB. The impact of obesity on outcomes of laparoscopic surgery for colorectal cancer in asians. Surg Endosc. (2010) 24(7):1679–85. 10.1007/s00464-009-0829-0 20039065

[11] Zhang H Hu H Huang R Guan Z Zheng M Xu C Natural orifice specimen extraction surgery versus conventional laparoscopic-assisted resection for colorectal cancer in elderly patients: a propensity-score matching study. Updat Surg. (2022) 74(2):599–607. 10.1007/s13304-021-01143-y 34370279

[12] Wexner SD. Further validation of the wexner incontinence score: a note of appreciation and gratitude. Surgery. (2021) 170(1):53–4. 10.1016/j.surg.2021.02.039 33863582

[13] Jorge JM Wexner SD. Etiology and management of fecal incontinence. Dis Colon Rectum. (1993) 36(1):77–97. 10.1007/BF02050307 8416784

[14] Dindo D Demartines N Clavien PA. Classification of surgical complications: a new proposal with evaluation in a cohort of 6336 patients and results of a survey. Ann Surg. (2004) 240(2):205–13. 10.1097/01.sla.0000133083.54934.ae 15273542 PMC1360123

[15] Cao Y He M Liu Z Chen K Denis K Zhang J Evaluation of the efficacy of natural orifice specimen extraction surgery versus conventional laparoscopic surgery for colorectal cancers: a systematic review and meta-analysis. Colorectal Dis. (2025) 27(1):e17279. 10.1111/codi.17279 39763245

[16] Steventon RD. A device for the female urinary outflow tract. Br J Radiol. (1985) 58(688):390. 10.1259/0007-1285-58-688-390-a 4063688

[17] Tuech JJ Regenet N Hennekinne S Pessaux P Bergamaschi R Arnaud JP. Laparoscopic colectomy for sigmoid diverticulitis in obese and nonobese patients: a prospective comparative study. Surg Endosc. (2001) 15(12):1427–30. 10.1007/s00464-001-9023-8 11965459

[18] Tsujinaka S Konishi F Kawamura YJ Saito M Tajima N Tanaka O Visceral obesity predicts surgical outcomes after laparoscopic colectomy for sigmoid colon cancer. Dis Colon Rectum. (2008) 51(12):1757–65. discussion 1765–1767. 10.1007/s10350-008-9395-0 18600376

[19] Hsu YJ Yu YL Jhuang JR You JF Liao CK Tsai WS Comparison of laparoscopic and open surgery for colorectal malignancy in obese patients: a propensity score-weighted cohort study. Int J Surg Lond Engl. (2024) 110(8):4598–607. 10.1097/JS9.0000000000001536 PMC11325910 38833348

[20] Panteleimonitis S Popeskou S Harper M Kandala N Figueiredo N Qureshi T Minimally invasive colorectal surgery in the morbid obese: does size really matter? Surg Endosc. (2018) 32(8):3486–94. 10.1007/s00464-018-6068-5 29362912 PMC6061053

[21] Leroy J Ananian P Rubino F Claudon B Mutter D Marescaux J. The impact of obesity on technical feasibility and postoperative outcomes of laparoscopic left colectomy. Ann Surg. (2005) 241(1):69–76. 10.1097/01.sla.0000150168.59592.b9 15621993 PMC1356848

[22] Scheidbach H Benedix F Hügel O Kose D Köckerling F Lippert H. Laparoscopic approach to colorectal procedures in the obese patient: risk factor or benefit? Obes Surg. (2008) 18(1):66–70. 10.1007/s11695-007-9266-0 18080169

[23] Kang J Baek SE Kim T Hur H Min BS Lim JS Impact of fat obesity on laparoscopic total mesorectal excision: more reliable indicator than body mass index. Int J Colorectal Dis. (2012) 27(4):497–505. 10.1007/s00384-011-1333-2 22065107

[24] Makino T Trencheva K Shukla PJ Rubino F Zhuo C Pavoor RS The influence of obesity on short- and long-term outcomes after laparoscopic surgery for colon cancer: a case-matched study of 152 patients. Surgery. (2014) 156(3):661–8. 10.1016/j.surg.2014.03.023 24947645

[25] Mao D Flynn DE Yerkovich S Tran K Gurunathan U Chandrasegaram MD. Effect of obesity on post-operative outcomes following colorectal cancer surgery. World J Gastrointest Oncol. (2022) 14(7):1324. 10.4251/wjgo.v14.i7.1324 36051092 PMC9305574

[26] Guan X Liu Z Longo A Cai JC Tzu-Liang Chen W Chen LC International consensus on natural orifice specimen extraction surgery (NOSES) for colorectal cancer. Gastroenterol Rep. (2019) 7(1):24–31. 10.1093/gastro/goy055 PMC6375350 30792863

[27] Yu YL Hsu YJ Liao CK Lin YC You JF Tsai WS Advantage of laparoscopic surgery in patients with generalized obesity operated for colorectal malignancy: a retrospective cohort study. Front Surg. (2022) 9:1062746. 10.3389/fsurg.2022.1062746 36684184 PMC9852741

[28] Zhang Q Wang M Ma D Zhang W Wu H Zhong Y Short-term and long-term outcomes of natural orifice specimen extraction surgeries (NOSES) in rectal cancer: a comparison study of NOSES and non-NOSES. Ann Transl Med. (2022) 10(8):488. 10.21037/atm-22-1175 35571383 PMC9096365

[29] Wang YLM Huang R Wu HY Hu HQ Jin YH Tang QC Totally laparoscopic resection and natural orifice specimen extraction surgery (NOSES) in synchronous rectal and gastric cancer. Gastroenterol Rep. (2020) 8(1):79–81. 10.1093/gastro/goz064 PMC7034228 32104588

[30] Zhu Y Xiong H Chen Y Liu Z Jiang Z Huang R Comparison of natural orifice specimen extraction surgery and conventional laparoscopic-assisted resection in the treatment effects of low rectal cancer. Sci Rep. (2021) 11(1):9338. 10.1038/s41598-021-88790-8 33927293 PMC8085046

[31] Senagore AJ Delaney CP Madboulay K Brady KM Fazio VW. Laparoscopic colectomy in obese and nonobese patients. J Gastrointest Surg. (2003) 7(4):558–61. 10.1016/S1091-255X(02)00124-5 12763416

