# Supplementary material for: Outcomes of high BMI colorectal cancer patients with natural orifice specimen extraction surgery: a propensity-score matching study
Source: Front Surg. 2025 Aug 21;12:1624266. doi: 10.3389/fsurg.2025.1624266 (PMC12410070; doi:10.3389/fsurg.2025.1624266)
Supplement: Supplementary file 1 [file Datasheet1.docx]

**Supplementary material**

**Table 1. perioperative outcomes of patients according to technique.**

| Characteristic | EXER(n=42) | EVER(n=26) | | IREX(n=20) |  | P | |  |
| --- | --- | --- | --- | --- | --- | --- | --- | --- |
| Operative time (min)^a^ | 204.74±57.0 | | 184.96±37.4 | 163.75±35.2 | |  | 0.734 | |
| Blood loss (ml) a | 55.36±64.40 | | 41.54±35.50 | 51.00±41.50 | |  | 0.296 | |
| Time to first flatus (h) a | 55.57±24.10 | | 43.86±21.90 | 48.30±25.10 | |  | 0.716 | |
| Time to first diet (h)a | 70.69±39.10 | | 63.92±28.40 | 68.90±29.50 | |  | 0.984 | |
| Postoperative hospital stays (days)a | 15.17±8.50 | | 13.54±7.90 | 10.40±2.70 | |  | 0.979 | |
| Positive margin | 0(0) | | 0(0) | 0(0) | |  | - | |
| Postoperative complication | 5(11.9) | | 1(3.8) | 1(5.0) | |  | 0.300 | |
| Harvested lymph nodesa | 12.70±3.80 | | 14.73±5.50 | 12.7±5.10 | |  | 0.208 | |
| Tumor grade |  | |  |  | |  |  | |
| Well-differentiated | 3(7.1) | | 3(11.5) | 3(15) | |  | 0.273 | |
| Moderately differentiated | 30(71.4) | | 22(84.6) | 15(75) | |  |  | |
| Poorly differentiated | 9(21.4) | | 1(3.8) | 2(10) | |  |  | |
| Histological type |  | |  |  | |  |  | |
| Adenocarcinoma | 32(76.2) | | 25(96.2) | 18(90) | |  | 0.214 | |
| Mucinous/Signet-ring cell | 1(2.4) | | 0(0) | 0(0) | |  |  | |
| Others | 9(21.4) | | 1(3.8) | 2(10) | |  |  | |

^a^ Mean±SD. Bold marked figures are for variables with P < 0.05.

**Fig.2 Survival comparisons. (A)Overall survival** (EXER vs. EVER P=0.281, EVER vs. IREX P=0.200, EVER vs. IREX P=0.495).**(B)Disease-free survival**(EXER vs. EVER P=0.091, EXER vs. IREX P=0.571, EVER vs. IREX P=0.356).
